# Genomic region associated with pod color variation in pea (*Pisum sativum*)

**DOI:** 10.1093/g3journal/jkab081

**Published:** 2021-03-15

**Authors:** Kenta Shirasawa, Kazuhiro Sasaki, Hideki Hirakawa, Sachiko Isobe

**Affiliations:** 1 Kazusa DNA Research Institute, Kisarazu, Chiba 292-0818, Japan; 2 Institute for Sustainable Agro-ecosystem Services (ISAS), Graduate School of Agricultural and Life Sciences, The University of Tokyo, Nishitokyo, Tokyo 188-0001, Japan

**Keywords:** double-digest restriction site-associated DNA sequencing, genetic mapping, genome sequence, Mendel’s genes, nanopore sequencing

## Abstract

Pea (*Pisum sativum*) was chosen as the research material by Gregor Mendel to discover the laws of inheritance. Out of seven traits studied by Mendel, genes controlling three traits including pod shape, pod color, and flower position have not been identified to date. With the aim of identifying the genomic region controlling pod color, we determined the genome sequence of a pea line with yellow pods. Genome sequence reads obtained using a Nanopore sequencing technology were assembled into 117,981 contigs (3.3 Gb), with an N50 value of 51.2 kb. A total of 531,242 potential protein-coding genes were predicted, of which 519,349 (2.8 Gb) were located within repetitive sequences (2.8 Gb). The assembled sequences were ordered using a reference as a guide to build pseudomolecules. Subsequent genetic and association analyses led to the identification of a genomic region that controls pea pod color. DNA sequences at this genomic location and transcriptome profiles of green and yellow pod lines were analyzed, and genes encoding 3' exoribonucleases were selected as potential candidates controlling pod color. The results presented in this study are expected to accelerate pan-genome studies in pea and facilitate the identification of the gene controlling one of the traits studied by Mendel.

## Introduction

Advances in the field of genomics, owing to the development of next-generation sequencing (NGS) technologies, have enabled us to identify gene sequences associated with genetic traits (Nguyen *et al.* 2019). The field of classical genetics was initiated by Gregor Mendel through the discovery of the laws of inheritance in pea (*Pisum sativum*). Mendel studied seven traits of pea (Von Mendel 1866; Ellis *et al.* 2011; Reid and Ross 2011), including seed shape, seed color, flower color, pod shape, pod color, flower position, and stem height. However, genes controlling only four of these traits, seed shape (Bhattacharyya *et al.* 1990), seed color (Armstead *et al.* 2007; Sato *et al.* 2007), flower color (Hellens *et al.* 2010), and stem height (Lester *et al.* 1997), have been identified to date, based on sequence variation in the pea genome. Given its diploid and autogamous nature, pea shows simple inheritance patterns, which is believed to have facilitated the discovery of the laws of inheritance. However, the pea genome is large (∼4.5 Gb) (Arumuganathan and Earle 1991; Greilhuber and Ebert 1994) and complex because of the presence of highly repetitive sequences (Murray *et al.* 1981; Macas *et al.* 2007). These characteristics of the pea genome interfere with its genomic analysis. 

To the best of our knowledge, two genome assemblies of pea are publicly available, one of which is a chromosome-level reference genome assembly, “Pisum_sativum_v1a,” of a French pea cultivar “Caméor” (Kreplak *et al.* 2019), while the other is a draft genome assembly of a pea cultivar “Gradus No 2” (ASM301357v1) released by the Earlham Institute, UK, under the GenBank assembly accession number GCA_003013575.1. The Pisum_sativum_v1a assembly consists of seven pseudomolecules (3.3 Gb) anchored to pea chromosomes, and 14,267 scaffold sequences (685.4 Mb) unassigned to any chromosome but potentially harboring 44,756 genes. In addition, repetitive sequences account for ca. 83% of this genome assembly. On the other hand, the ASM301357v1 assembly (4.3 Gb) consists of as many as 5.4 million scaffold sequences; however, no annotation information is available. These two reference genome assemblies could help identify all genes and genetic variations present within *P. sativum* (Gan *et al.* 2011).

Pan-genomes comprise core genes found in all individuals within a species, in addition to dispensable genes present in only some accessions (Bayer *et al.* 2020). However, repetitive sequences interfere with the alignment of sequence reads to the reference genome assembly, decreasing the accuracy of pan-genome studies. Instead of sequence similarity searches, genetic anchoring of multiple reference sequences with DNA markers would provide more accurate pan-genome results. NGS-based genotyping techniques (Davey *et al.* 2011), such as double-digest restriction site-associated DNA sequencing (ddRAD-Seq; Peterson *et al.* 2012), would be effective for the comparative analysis of genome sequences and structures. For instance, a set of ddRAD-Seq reads obtained from a mapping population could be aligned to each of the multiple reference sequences for detecting single nucleotide polymorphisms (SNPs), and subsequent linkage analysis of these SNPs could cluster SNPs located at a given locus at a single map position (Shirasawa and Kitashiba 2017). This approach would be useful for genetically assignment of a sequence from one line to those from other lines.

In this study, we aimed to identify the genomic region that determines the pod color in pea. The genome of a pea line with yellow pods was sequenced, and the genome assembly was anchored to the publicly available reference sequences using SNPs obtained by ddRAD-Seq as anchors. Because the ddRAD-Seq data were derived from an F2 mapping population with segregating pod color and flower color, the color loci were genetically and physically identified by genetic mapping and genome-wide association study (GWAS) approaches. Subsequent whole-genome resequencing and transcriptome analyses identified candidate genes controlling pod color and flower color in pea. Thus, the results of this study not only facilitate pan-genomic studies in pea but also enable the discovery of genes controlling traits studied by Mendel.

## Materials and methods

### Plant material

Two pea lines, JI4 (white flowers and green pods) and JI128 (red flowers and yellow pods), provided by John Innes Centre (Norwich, UK), were used in this study. Line JI4 carries recessive alleles for flower color (*a*) and dominant alleles for pod color (*Gp*), whereas line JI128 possesses recessive alleles for pod color (*gp*). The two pea lines were crossed to obtain F1 seeds. F2 plants (*n *=* *167) were grown in an experimental field at the Institute for Sustainable Agro-ecosystem Services, Graduate School of Agricultural and Life Sciences, The University of Tokyo (35°74′N, 139°54′E), and flower and pod colors were visually evaluated.

### *De novo* genome assembly

DNA was extracted from the leaves of JI128 plants using the DNeasy Plant Mini Kit (Qiagen, Hilden, Germany). Short-read DNA sequencing libraries were prepared according to the manufacturer’s instructions, and sequenced on DNA sequencers, NextSeq 500 (Illumina) and DNBSEQ-G400 (MGI Tech, Shenzhen, China). The genome size of line JI128 was estimated by *k*-mer distribution analysis using the Jellyfish software (Marcais and Kingsford 2011).

High-molecular-weight DNA was extracted from JI128 DNA using the Genomic-tip kit (Qiagen), and a long-read sequence library was constructed using the Rapid Sequencing Kit (version SQK-RAD004) (Oxford Nanopore Technologies, Oxford, UK). The library was sequenced with the MinION using flow cell version FLO-MIN107 R9 (Oxford Nanopore Technologies). Base calling from the FAST5 files was performed using Guppy v2.3.5 (Oxford Nanopore Technologies). Long reads were assembled with wtdbg2 v2.2 (Ruan and Li 2020), and potential sequencing errors in the contigs were corrected only once using Pilon (Walker *et al.* 2014). The resulting genome assembly was designated as PSA_r1.0. Assembly completeness was evaluated with the embryophyta_odb10 data using Benchmarking Universal Single-Copy Orthologs (BUSCO) v3.0.2 (Simao *et al.* 2015).

### Repetitive sequence analysis and gene prediction

JI128 contigs were aligned to the pseudomolecule sequences of Pisum_sativum_v1a (Kreplak *et al.* 2019) with RaGOO v.1.1 (Alonge *et al.* 2019) to establish chromosome-level sequences. Repetitive sequences were detected with RepeatMasker v.4.1.1 (http://www.repeatmasker.org) using repeat sequences obtained from the JI128 sequence assembly with RepeatModeler v.2.0.1 (http://www.repeatmasker.org) and from a dataset registered in Repbase (Bao *et al.* 2015). Potential protein-coding genes were predicted by an ab-initio method of Augustus v.3.3.3 (Stanke *et al.* 2006), with the training dataset obtained from BUSCO analysis. Overlapping sequences of repeats and predicted genes were detected with the intersect command of BEDtools v.2.29.1 (Quinlan and Hall 2010).

### ddRAD-Seq analysis

The genomic DNA of 168 F2 lines, an F1 hybrid, and both parental lines (JI4 and JI128) were subjected to ddRAD-Seq analysis. The ddRAD-Seq libraries were prepared using two restriction enzymes, *Pst*I and *Msp*I, as described previously (Shirasawa *et al.* 2016), and sequenced on the HiSeq4000 platform (Illumina). Low-quality bases were removed from the reads using PRINSEQ v0.20.4 (Schmieder and Edwards 2011), and adapter sequences were trimmed using fastx_clipper in the FASTX-Toolkit v0.0.13 (http://hannonlab.cshl.edu/fastx_toolkit). Bowtie2 v2.2.3 (Langmead and Salzberg 2012) was used to map the filtered reads onto three reference genome sequence assemblies: Pisum_sativum_v1a (Kreplak *et al.* 2019), ASM301357v1 (GenBank accession number PUCA000000000), and PSA_r1.0 (this study). The resultant Sequence Alignment/Map (SAM) files were converted to Binary Sequence Alignment/Map (BAM) files and subjected to SNP calling using the mpileup option of SAMtools v0.1.19 (Li *et al.* 2009) and the view option of BCFtools. High-confidence SNPs were selected using VCFtools v0.1.12b (Danecek *et al.* 2011), according to the following criteria: (1) depth of coverage ≥5 (for each line); (2) SNP quality score = 999 (for each locus); and (3) proportion of missing data <0.5 (for each locus).

### Genetic map construction

Linkage analysis of high-confidence SNPs was performed with LepMap3 v0.2 (Rastas 2017). Marker loci were classified roughly into seven linkage groups using the SeparateChromosomes2 module (logarithm of the odds [LOD] score = 19), while considering segregation distortion. Marker order and map distances were calculated using the OrderMarkers2 module. The pod color trait was mapped on the resultant genetic map with MAPMAKER v3.0b (Lander *et al.* 1987).

Association mapping was performed using the general linear model implemented in TASSEL version 5.0 (Bradbury *et al.* 2007). Thresholds for the association were set at a significance level of 1%, after Bonferroni multiple test correction.

### Whole genome resequencing analysis

Short-read sequence libraries for JI4 and JI128 were prepared using the TruSeq DNA PCR-Free Library Kit (Illumina), according to the manufacturer’s instructions, and sequenced on the NextSeq500 system (Illumina). Low-quality bases and adapter sequences were trimmed and mapped onto the three reference genome assemblies, as described above. High-confidence SNPs were selected using VCFtools v0.1.12b (Danecek *et al.* 2011), according to the following criteria: (1) depth of coverage ≥ 5 (for each line); (2) SNP quality score = 999 (for each locus); and (3) proportion of missing data <0.5 (for each locus). Effects of SNPs on gene function were predicted using SnpEff v4.3t (Cingolani *et al.* 2012).

### RNA-Seq analysis

Total RNA was extracted from immature pods of JI4 and JI128 using the RNeasy Mini Kit (Qiagen). The isolated total RNA was treated with RQ1 RNase-Free DNase (Promega, Madison, WI, USA) to remove contaminating genomic DNA, and RNA libraries were constructed using the TruSeq Stranded mRNA Library Kit (Illumina). The RNA libraries were sequenced on the NextSeq 500 System (Illumina) to generate 151 bp paired-end reads. Low-quality bases were removed with PRINSEQ v0.20.4 (Schmieder and Edwards 2011), and adapter sequences were trimmed with fastx_clipper (parameter: ‐a AGATCGGAAGAGC) in the FASTX‐Toolkit v0.0.13 (http://hannonlab.cshl.edu/fastx_toolkit). Gene expression was quantified by mapping the RNA-Seq reads onto the PSA_r1.0 assembly using HISAT2 v2.1.0 (Kim *et al.* 2015), followed by sequencing depth normalization to determine the number of fragments per kilobase of exon model per million mapped reads (FPKM) using StringTie v1.3.5 (Pertea *et al.* 2015) and Ballgown v2.14.1 (Frazee *et al.* 2015), as described previously (Pertea *et al.* 2016).

### Data availability

The sequence reads were deposited to the Sequence Read Archive (SRA) database of the DNA Data Bank of Japan (DDBJ) under the accession numbers DRA010800 and DRA010801. The DDBJ accession numbers of the assembled contig sequences used to generate the PSA_r1.0 genome sequence assembly are BNEU01000001–BNEU01117981. The genome sequence information of JI128 is available from Plant GARDEN (https://plantgarden.jp). Supplemental Material available at figshare: https://doi.org/10.25387/g3.14188685.

## Results

### *De novo* genome assembly of JI128 possessing yellow pod color

**Figure 1:**
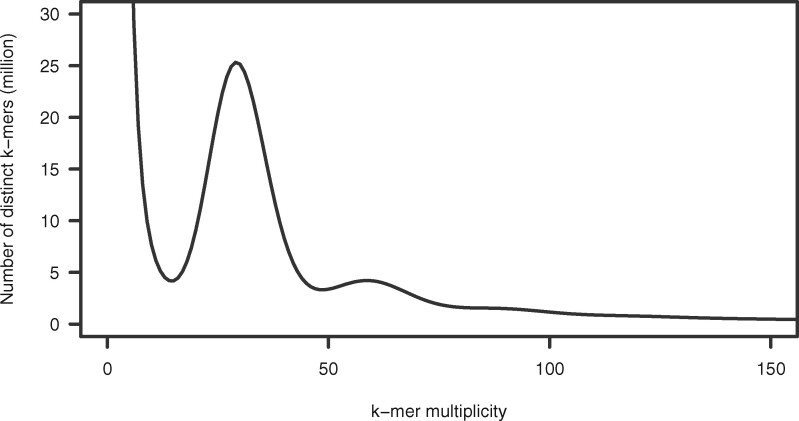
Genome size estimation for the pea line, JI128, based on the distribution of the number of distinct *k*-mers (*k* = 17), with the given multiplicity values.

The genome sequence of JI128 was determined using the Rapid Sequencing Kit (version SQK-RAD004), a long-read sequencing technology of Oxford Nanopore Technologies (Oxford, UK). First, the size of the JI128 genome was estimated as 4.5 Gb via *k*-mer distribution analysis of the short-read data (147 Gb) obtained from DNBSEQ-G400 (Figure 1, Supplementary Table S1). The library was sequenced on 35 flow cells with the MinION, and a total of 262.1 Gb data were obtained, consisting of 33.2 million (M) reads with an N50 read length of 15.5 kb (Supplementary Table S1). Potential sequencing errors were corrected, and the cleaned reads (total 12.3 M reads; 196.7 Gb; 44× genome coverage) were assembled into 117,981 contigs. BUSCO analysis indicated only 51.0% of the complete BUSCOs in the assembly. Then, sequencing errors in the assembly were corrected using Pilon, for which Illumina and MGI short reads were employed. The final assembly was 3297 Mb in length, with an N50 value of 51.2 kb; this assembly was designated as PSA_r1.0 (Table 1). Although the sequence length (3.3 Gb) was 26.7% (1.2 Gb) shorter than the expected size (4.5 Gb), 95.4% of the complete BUSCOs were represented in the contigs (Table 1).

**Table 1:** Statistics of the genome assemblies of pea lines

	PSA_r1.0	ASM301357v1	Pisum_sativum_v1a
Pea line	JI128	Gradus No 2	Caméor
Total contig size (bp)	3,297,037,789	4,274,305,877	3,159,358,344
Number of contigs	117,981	5,465,676	218,010
Contig N50 length (bp)	51,222	4,601	37,931
Complete BUSCOs	95.4%	91.5%	Not available
Single-copy BUSCOs	93.2%	88.8%	Not available
Duplicate BUSCOs	2.2%	2.7%	Not available
Fragmented BUSCOs	1.7%	4.6%	Not available
Missing BUSCOs	2.9%	3.9%	Not available
Reference	This study	GCA_003013575.1^a^	Kreplak *et al.* (2019)

a GenBank assembly accession number.

Of the 117,981 contigs, 116,071 sequences (3276 Mb) were aligned to the pseudomolecule sequences of Pisum_sativum_v1a (Kreplak *et al.* 2019) and concatenated with 100 Ns to establish seven chromosome-level sequences, PSA_r1.0.pmol. The remaining 1910 contigs (20.6 Mb) were also concatenated to generate chromosome 0 and included in PSA_r1.0.pmol. Repetitive sequences were identified in 2825 Mb (85.7%) of the PSA_r1.0.pmol, including LTR retrotransposons, which occupied 2284 Mb of PSA_r1.0.pmol. A total of 531,242 potential protein-coding genes were predicted in PSA_r1.0.pmol, of which 519,349 (97.8%) and 11,893 (2.2%) genes were located inside and outside of the repetitive regions, respectively, both of which would include functional or at least transcribed genes.

Short-read data of JI128 (80.3 Gb) and JI4 (68.7 Gb) obtained using the Illumina NextSeq 500 System (Supplementary Table S1) were mapped onto the PSA_r1.0, Pisum_sativum_v1a, and ASM301357v1 assemblies, leading to the identification of 6,374,054, 6,048,006, and 6,152,891 high-confidence SNPs, respectively, between the two lines.

### Genetic map construction

**Figure 2:**
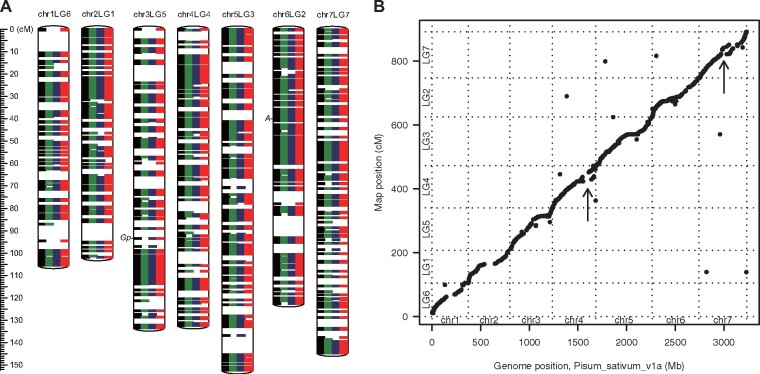
Genetic linkage map of pea. (A) Genetic maps of PSA_r1.0, Pisum_sativum_v1a, and ASM301357v1 genome sequence assemblies. Green, blue, and red colors indicate genetic bins detected in PSA_r1.0, Pisum_sativum_v1a, and ASM301357v1 genome assemblies, respectively, and black bars indicate bins common to the three assemblies. The genetic loci for flower color and pod color are indicated as *A* and *Gp*, respectively. (B) Dot plot showing the collinearity between the genetic map (*y*-axis) and physical map of Pisum_sativum_v1a (*x*-axis). Arrows indicate potential chromosomal rearrangements.

**Figure 3:**
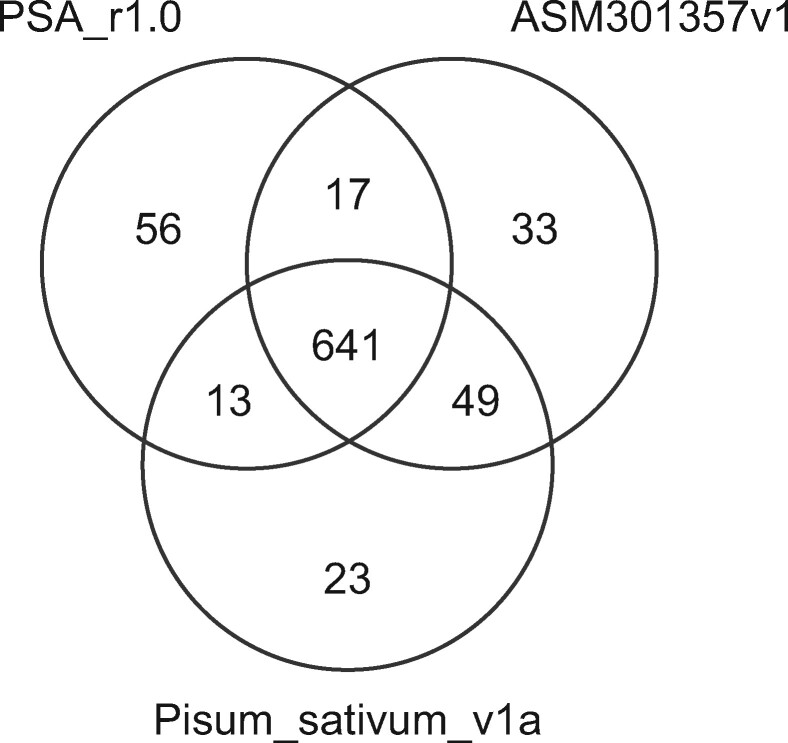
Number of genetic bins detected in the three reference genome assemblies, PSA_r1.0, Pisum_sativum_v1a, and ASM301357v1. The number of bins common or unique to the genome assemblies are shown by a Venn diagram.

A genetic map was constructed based on SNPs identified by ddRAD-Seq analysis of the F2 population, F1 hybrid, and parental lines. A total of 2.4 M reads were obtained per sample using ddRAD-Seq (Supplementary Table S1), of which 95.1% were mapped onto the PSA_r1.0 assembly. High-confidence SNPs were selected from 2183 loci located on 939 contigs. The ddRAD-Seq reads were mapped in parallel onto two publicly available pea genome sequence assemblies, ASM301357v1 and Pisum_sativum_v1a, with mapping rates of 95.3% and 95.4%, respectively, leading to the identification of 2304 and 2246 SNPs, respectively. Out of a total of 6733 data points (= 2183 + 2304 + 2246), 6238 were separated into seven linkage groups and ordered. The linkage groups were named according to Kreplak *et al.* (2019). The resultant 891.8-cM genetic map contained 6023 data points assigned to 832 genetically independent bins (Figure 2A, Table 2, Supplementary Table S2). These SNPs mapped to 1998, 2034, and 1991 loci (727, 740, and 726 bins, respectively) on the PSA_r1.0, ASM301357v1, and Pisum_sativum_v1a reference genome assemblies, respectively. While 641 bins were common to all three reference genome assemblies, 56, 33, and 23 bins were unique to PSA_r1.0, ASM301357v1, and Pisum_sativum_v1a, respectively (Figure 3).

**Table 2:** Genetic map length and number of single nucleotide polymorphisms (SNPs) and genetic bins

Linkage group	Integrated			PSA_r1.0			ASM301357v1			Pisum_sativum_v1a		
	No. of SNPs	No. of genetic bins	Map length (cM)	No. of SNPs	No. of genetic bins	Map length (cM)	No. of SNPs	No. of genetic bins	Map length (cM)	No. of SNPs	No. of genetic bins	Map length (cM)
chr1LG6	576	89	105.4	197	80	94.8	191	79	105.4	188	74	94.8
chr2LG1	758	104	101.9	257	95	101.3	255	92	101.9	246	87	101.9
chr3LG5	788	109	133.2	258	95	128.7	262	98	133.2	268	99	128.7
chr4LG4	774	116	132.2	264	104	131.9	252	100	132.2	258	98	131.9
chr5LG3	1,257	151	152.3	413	128	152.3	434	128	152.3	410	137	152.3
chr6LG2	1,020	135	122.4	330	115	121.2	350	127	122.4	340	119	122.4
chr7LG7	850	128	144.4	279	110	144.4	290	116	144.4	281	112	144.4
Total	6,023	832	891.8	1,998	727	874.6	2,034	740	891.8	1,991	726	876.4

On the basis of the genetic map, 918 contig sequences of PSA_r1.0 (135.8 Mb) and 1001 contigs of ASM301357v1 (31.2 Mb) were anchored to the chromosomes (Supplementary Table S2); however, the total lengths of the assigned sequences were quite shorter than the estimated genome size. In Pisum_sativum_v1a, on the other hand, 91 scaffold and super-scaffold sequences (19.0 Mb) that had not been integrated into the seven pseudomolecule sequences (chr1LG6 to chr7LG7; 3.2 Gb) were newly assigned to pea chromosomes. Subsequently, we examined collinearity between the genetic and physical maps of Pisum_sativum_v1a (Figure 2B). The results indicated that while most of the genome structures were conserved between the two maps, there might be potential large-scale differences between chr4G4 and chr7LG7 because of the disruption of the collinearity.

### Flower color gene at the *A* locus

Of the 167 plants in the F2 population, 109 produced red flowers and 44 produced white flowers (Figure 1); the remaining 14 plants were not evaluated for the flower color. Thus, the white: red segregation ratio fit the Mendelian segregation ratio for a single gene (*p *=* *0.283; χ^2^ = 1.153). In the F1 progeny, red flower color was dominant to white flower color.

The flower color trait was mapped to a single locus on chr6LG2 (39.8 cM) of the genetic map (Figure 2A). This mapping result was supported by GWAS (*p *=* *2.9 × 10^−77^). The physical location of this locus corresponded to 25,106 bp on Psa1.0_019194.1 (PSA_r1.0), 1423 bp on PUCA013577394.1 (ASM301357v1), and 68,269,988 bp on chr6LG2 (Pisum_sativum_v1a). The SNP responsible for flower color variation in pea was located ∼60 kb away from the Psat6g060480 gene (*A*), which encodes a basic helix-loop-helix (bHLH) protein (Hellens *et al.* 2010). The SNP (adenine in JI4 and guanine in JI128) presumably responsible for flower color variation in pea was detected at the splice donor site of the 6th intron (at 68,336,837 bp on chr6LG2), based on the whole genome resequencing data of JI4 and JI128.

### Pod color gene candidates at the *Gp* locus

All F1 plants produced green pods. However, in the F2 population, 126 plants produced green pods, while 38 plants produced yellow pods (Figure 1); three lines had no pods at the time of phenotyping and therefore could not be evaluated for pod color. Thus, the pod color ratio fit the Mendelian segregation ratio for a single gene (*p *=* *0.589; χ^2^ = 0.293). Together, these data indicate that pea pod color is controlled by a single gene, and green pod color is dominant to yellow pod color.

The pod color trait was genetically mapped to a single locus on chr3LG5 at 93.3 cM (Figure 2A). This map position was close to the *Gp* locus studied by Mendel (Ellis and Poyser 2002; Ellis *et al.* 2011). The GWAS approach detected this position (*p *=* *2.9 × 10^−117^) at 17,394 bp on scaffold04355 (Pisum_sativum_v1a) and at 1901 bp on PUCA010639216.1 (ASM301357v1); both these sequences have not been physically assigned to pea chromosomes.

**Figure 4:**
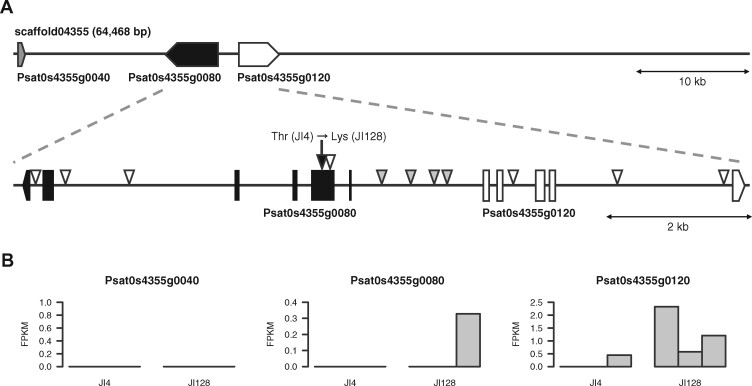
Genomic and transcriptomic analyses of scaffold04355 of Pisum_sativum_v1a. (A) Genome structure of scaffold04355. Gray, black, and white boxes represent the Psat0s4355g0040, Psat0s4355g0080, and Psat0s4355g0120 genes, respectively. Triangles represent sequence variations between JI128 and JI4; white, black, and gray triangles represent silent, non-synonymous, and regulatory variants, respectively. (B) Transcriptome-based RNA expression patterns of genes in scaffold04355. Bars indicate the expression levels of genes in three replicates of immature pods of JI128 and JI4, expressed as fragments per kilobase of exon model per million mapped reads (FPKM).

While the sequence length of PUCA010639216.1 was only 5818 bp, that of scaffold04355 was 64,468 bp, and three genes were predicted on scaffold04355, including Psat0s4355g0040, Psat0s4355g0080, and Psat0s4355g0120 (Figure 4). Psat0s4355g0040 was annotated as an unknown gene, while Psat0s4355g0080 and Psat0s4355g0120 were predicted as members of the 3’ exoribonuclease gene family. Interestingly, Psat0s4355g0080 and Psat0s4355g0120 were arranged head-to-head, with an interval of approximately 1.8 kb. Since these genes were potential candidate genes controlling the pod color trait, comparative genome and transcriptome analyses of JI4 and JI128 were performed.

Whole genome resequencing analysis revealed a total of 36 sequence variations (28 SNPs and eight indels) between JI4 and JI128 on scaffold04355. Of these, only one SNP was a non-synonymous mutation in the second exon of Psat0s4355g0080, while the remaining 35 polymorphisms were silent mutations (six intronic variants, 28 intergenic variants, and one synonymous mutation) (Figure 4). Among the intergenic variants, two SNPs and two indels were located on the region between Psat0s4355g0080 and Psat0s4355g0120, which probably corresponded to the promoter regions of the two genes (Figure 4).

To analyze gene expression, three replicates of immature pods of each line were investigated by RNA-Seq. Approximately 21.2 M high-quality reads were obtained from each sample (Supplementary Table S1) and mapped onto the Pisum_sativum_v1a reference genome assembly with a mapping rate of 93.9%. Out of 44,756 genes predicted in Pisum_sativum_v1a (Kreplak *et al.* 2019), 25,726 genes were expressed in at least one of the six samples. The expression level of Psat0s4355g0120 in JI128 (1.4) was approximately 10-fold higher than that in JI4 (0.15) (Figure 4). No obvious differences were detected in the expression levels of Psat0s4355g0040 and Psat0s4355g0080 between the two pea lines; Psat0s4355g0040 expression was not detected in either line, and Psat0s4355g0080 expression was detected in only one of the three replicates of JI128.

## Discussion

In this study, we report the genome sequence assembly of the pea line, JI128, possessing yellow pods. Contigs were genetically anchored to the publicly available pea reference genome assemblies, Pisum_sativum_v1a and ASM301357v1. Our analysis provides information on the common genetic loci across the different genome sequence assemblies. Furthermore, the locus associated with pod color was identified on the genetic map, and the genes underlying this locus were identified on the reference genome sequence assemblies. Genomic and transcriptomic analyses revealed candidate genes responsible for the differences in pod color between JI128 and JI4.

To construct the genome sequence assembly of JI128 (PSA_r1.0), we employed a long-read sequencing technology of Oxford Nanopore Technologies (44× genome coverage). The PSA_r1.0 assembly showed higher contiguity (N50 = 51,222 bp) than the ASM301357v1 assembly (N50 = 4601 bp), which was generated from Illumina short-read sequences of a single library (86× genome coverage), and similar to the Pisum_sativum_v1a assembly (N50 = 37,931 bp), which was constructed using Illumina short reads of multiple insert-size libraries (281× genome coverage) (Kreplak *et al.* 2019; Table 1). However, the contiguity of assembled sequences obtained in this study was lower than expected. No contig sequences for the pod color locus were represented in the PSA_r1.0 assembly. It might be necessary to extend the sequences by integrating whole genome profiles of a bacterial artificial chromosome (BAC) library and/or an optical mapping technology of BioNano Genomics, as reported previously (Kreplak *et al.* 2019). Alternatively, a PacBio long-read technology, which produces high-quality long reads known as HiFi reads, might improve the assembly (Wenger *et al.* 2019; Lang *et al.* 2020).

The PSA_r1.0 assembly, together with contigs of ASM301357v1, was anchored to the pseudomolecule sequences of Pisum_sativum_v1a (Supplementary Table S2). With the genetic map based on the ddRAD-Seq analysis, 918 and 1001 contigs of PSA_r1.0 and ASM301357v1, respectively, were genetically assigned to the pseudomolecule sequences of Pisum_sativum_v1a, despite the short lengths of the anchored sequences (PSA_r1.0: 135.8 Mb; ASM301357v1: 31.2 Mb), because of the low contiguity of the assemblies. Hence, we employed the reference-based method to construct the pseudomolecule sequences of JI128 that spanned a 3.3 Gb sequence. Thus, it is important to improve the *de novo* assembly contiguity and/or increase the number of genetic loci for covering most of the contig sequences. Whole genome resequencing of mapping populations (Mascher *et al.* 2013) has become a realistic alternative to ddRAD-Seq, owing to the reduction in the cost of sequencing. Given the potential for large-scale chromosomal rearrangements in the reference genome (Figure 2B), in addition to the unassigned contig sequences, the genetic anchoring approach would be more useful than the reference-guided methods for genetic analyses in the pan-genome era (Bayer *et al.* 2020).

The 3’ exoribonuclease protein encoding genes, Psat0s4355g0080 and Psat0s4355g0120, were identified as potential candidates for the pod color. Psat0s4355g0120 was expressed in JI128, while Psat0s4355g0080 showed a missense mutation. Therefore, both these genes are currently considered as potential candidates for pod color. While the 3’ exoribonuclease proteins are reported to mediate mRNA degradation in Arabidopsis (Nguyen *et al.* 2015), the function of these genes has not yet been analyzed in pea. We propose a hypothetical scenario in which these genes lead to the formation of yellow pods. The 3' exoribonuclease family proteins might suppress the production of a green pigment, probably chlorophyll, in pods or degrade the products, for example, in the green cotyledons of pea (Armstead *et al.* 2007; Sato *et al.* 2007). However, while the yellow color was dominant to the green color in cotyledons, an opposite trend was observed in pods. Therefore, the gene responsible for pod color would not correspond to genes previously reported to regulate chlorophyll breakdown. Since the 3' exoribonuclease family proteins form dimers (Zuo and Deutscher 2001), this dimerization might be required to suppress the factors involved in the biosynthesis of the green pigment. In heterozygous plants, if the protein dimer contained one subunit encoded by the dominant allele and the other subunit encoded by the recessive allele, the resulting dimer might lose function in accordance with the dominant-negative effect (Veitia 2007), which would explain the results obtained in this study. However, further investigation is needed to verify this hypothesis and to identify the gene for the green pod color.

In conclusion, we determined the genome sequence of a pea line, JI128, possessing yellow pods, and identified the genetic locus responsible for the pea pod color. Since the map position of the pod color locus was closed to the locus reported by Mendel, we predict that the identified genomic region contains the gene responsible for the pod color of pea. Thus, the results of this study facilitate the identification of the gene controlling one of the seven traits studied by Mendel (Ellis and Poyser 2002; Ellis *et al.* 2011) and are expected to facilitate pan-genome studies in pea.
